# High Phylogenetic Diversity of Glycosyl Hydrolase Family 10 and 11 Xylanases in the Sediment of Lake Dabusu in China

**DOI:** 10.1371/journal.pone.0112798

**Published:** 2014-11-13

**Authors:** Guozeng Wang, Xiaoyun Huang, Tzi Bun Ng, Juan Lin, Xiu Yun Ye

**Affiliations:** 1 College of Biological Science and Technology, Fuzhou University, Fuzhou 350108, P.R. China; 2 National Engineering Laboratory for High-efficiency Enzyme Expression, Fuzhou 350002, P. R. China; 3 School of Biomedical Sciences, Faculty of Medicine, The Chinese University of Hong Kong, Shatin, New Territories, Hong Kong, China; USDA/ARS, United States of America

## Abstract

Soda lakes are one of the most stable naturally occurring alkaline and saline environments, which harbor abundant microorganisms with diverse functions. In this study, culture-independent molecular methods were used to explore the genetic diversity of glycoside hydrolase (GH) family 10 and GH11 xylanases in Lake Dabusu, a soda lake with a pH value of 10.2 and salinity of 10.1%. A total of 671 xylanase gene fragments were obtained, representing 78 distinct GH10 and 28 GH11 gene fragments respectively, with most of them having low homology with known sequences. Phylogenetic analysis revealed that the GH10 xylanase sequences mainly belonged to Bacteroidetes, Proteobacteria, Actinobacteria, Firmicutes and Verrucomicrobia, while the GH11 sequences mainly consisted of Actinobacteria, Firmicutes and Fungi. A full-length GH10 xylanase gene (*xynAS10-66*) was directly cloned and expressed in *Escherichia coli*, and the recombinant enzymes showed high activity at alkaline pH. These results suggest that xylanase gene diversity within Lake Dabusu is high and that most of the identified genes might be novel, indicating great potential for applications in industry and agriculture.

## Introduction

Xylan is the major hemicellulose component of the plant cell wall, which is the second most abundant polysaccharide on earth after cellulose [1]. Xylan is composed of a homopolymeric backbone chain of β-1, 4-linked xylopyranose units with substituted side chains at different positions, and its complete hydrolysis requires a group of enzymes including endo-1,4-β-D-xylanase, β-D-xylosidase, α-D-glucuronidase, α-L-arabinofuranosidase, acetyl xylanesterase, and arylesterase [2], [3]. Endo-1,4-β-D-xylanase (EC 3.2.1.8) is a crucial component because it catalyzes the hydrolysis of xylan to short xylooligosaccharides of varying lengths [2], [3]. Xylanases have been classified into glycosyl hydrolase (GH) families (http://www.cazy.org/fam/acc_GH.html; [4]) 5, 7, 8, 10, 11 and 43 [3] based on sequence similarities of the catalytic domain. Among these, GH10 and 11 xylanases are the most abundant, which have distinct three-dimensional structures [5], mechanisms of action [6] and substrate specificity to xylan.

Although xylanases are produced by diverse organisms, including bacteria, algae, fungi, protozoans, gastropods and anthropods [3], microbial xylanases are the focus of intense research owing to their significant application in various industrial processes. Specifically, they are used to improve the digestibility of animal feedstock, enhance filterability in brewing, increase dough volume and improve the textural and staling properties of bread, and for fruit juice and wine clarification, the bioconversion of lignocellulosic materials into fermentative products, and facilitation of the release of lignin from the pulp [1], [2], [7], [8]. Many microbial xylanases have been purified and characterized, and the genes encoding xylanases have been cloned and expressed in heterologous systems, and the structures of a number of enzymes have been determined [3], [8].

Most microbial xylanases reported to date have acidic or neutral pH optima. Alkaline xylanases are of great interest because xylan is more readily soluble under alkaline pH than neutral pH. Additionally, the application of alkaline xylanases in the paper and pulp industry can reduce the use of chlorine, which is very attractive from an economical and technical point of view [9], [10]. Consequently, studies are continually conducted in attempts to identify novel xylanases with potential applications in the pulp and paper industries. Although alkaline xylanases have been reported from microorganisms isolated from nonalkaline environments [11], [12], xylanases produced by microorganisms isolated from extreme alkaline environments have attracted increasing attention. These organisms are exposed to hostile environments, resulting in their evolution and accumulation of a variety of adaptive features for activity and stability under these conditions [13]–[15].

Natural alkaline environments are not common, and soda lakes and deserts are the most stable naturally occurring alkaline ecosystems on earth [16], [17]. Soda lakes have high carbonate alkalinity, a pH of 9 to 11, and moderate to extremely high salinity [17]. Despite their extreme environmental conditions, soda lakes harbor extremely productive microbial communities. A number of alkaliphilic microorganisms that play key metabolic roles in soda lakes have previously been studied, and many novel alkaliphilic microorganisms have been isolated from these unique ecosystems [18]. Molecular biological techniques that do not depend on culture have revealed a high diversity and novelty of microbial communities in soda lake environments [19]–[21].

Although the microbial diversity of soda lakes has been studied extensively, the functional diversity of genes in such systems has not. Lin et al. analyzed the methane monooxygenase genes in Mono Lake and suggested that increased methane oxidation activity was correlated with changes in methanotroph community structure [22]. Kovaleva et al. explored the diversity of RuBisCO and ATP citrate lyase genes in the sediments from six soda lakes of the Kulunda Steppe [23]. However, few studies have focused on the functional gene diversity of plant material dehygrolysis in soda lake environments. We explored the genetic diversity of GH10 xylanase in diverse soil environments using culture-independent molecular methods and found that pH was one of the most important factors influencing the xylan degrading microbial community [24].

In this study, we focused on the genetic diversity of GH10 and GH11 xylanases in the natural Lake Dabusu, an alkaline (pH 10.2) and saline (101 g/liter) lake situated in northeastern China. Partial xylanase genes were amplified directly from the metagenomic DNA of the soda lake sediment. Sequence analysis showed that most xylanase gene fragments had low homologies to known xylanases, suggesting a large number of uncharacterized xylanase genes in the soda lake. Additionally, phylogenetic diversity analysis suggested a surprising diversity of xylanase genes in this harsh environment. A novel GH10 full-length xylanase gene was directly cloned from the metagenomic DNA and expressed in *Escherichia coli*. The recombinant xylanases showed high activity at alkaline pH. Our study provides new insight into the genetic diversity and distribution of microbial xylanases in the soda lake ecosystem, which will help us understand their roles in this microenvironment.

## Materials and Methods

### Ethics statement

We declare that no living animals were used in this research. No specific permissions were required for the described field studies. The sediment sample was collected from the location that is not privately-owned or protected in any way. The field studies did not involve endangered or protected species because this study only concentrated on the sediment sample.

### Sample site

Lake Dabusu is located in the southwestern part of the Qian'an County (Jilin Province, China), in the center of the depressed belt of Songliao Basin. Lake Dabusu is a closed inland alkaline lake located 122 meters above sea level, with an average water depth of about 0.90 meters and an area of approximately 38 km^2^ in the rainy season. Because of strong evaporation and a lack of outflow within the closed basin, alkaline materials accumulate in the lake water. Lake Dabusu is a typical soda lake with salinity of 62.34 g L^−1^ to 347.34 g L^−1^ and a pH of 10 to 11 depending on season [25].

### Sample collection and DNA extraction

On June 12, 2013, superficial (0 to 10 cm depth) sediment samples were collected and transported into a sterile sampling bag and immediately shipped to the laboratory on ice packs. Upon arrival, portions of the samples were stored at 4°C for physicochemical characterization and −80°C for metagenomic DNA extraction. Details regarding the location and physicochemical properties of the lake sediment are provided in Table 1. By using a modified protocol specific for high molecular weight DNA from environmental samples [26], the metagenomic DNA of the sediment was extracted as described in detail previously [27]. The sediment metagenomic DNA was purified using an Omega Gel Extraction Kit (Norcross, GA) and stored at −20°C until use.

**Table 1:** Physicochemical characters of the alkaline saline lake sediment and xylanase fragment sequences obtained.

Location	T (°C)	pH	Total organic carbon (mg/g)	Total nitrogen (mg/g)	C/N ratio	GH family	Clones sequenced	Sequences recovered	OTUs^a^
44 48 20 N	20	10.2	1.10	0.35	3.14	GH10	550	467	78
123 40 32 E						GH11	250	204	28

a 5% dissimilarity as cutoff.

### Xylanase gene fragments amplification, library construction and sequencing

GH10 and GH11 xylanase gene fragments were amplified with the purified metagenomic DNA as a template by touchdown PCR using the CODEHOP primers X10-F and X10-R specific for GH10 xylanase and X11-F and X11-R specific for GH11 xylanase [27], respectively. PCR products were then visualized on an agarose gel and purified using a Qiaquick gel extraction kit (Qiagen, Valencia, CA). The purified PCR products were then ligated into the PMD 19-T vector (TaKaRa, Tokyo, Japan) and electroporated into *Escherichia coli* DH5a (TaKaRa, Tokyo, Japan) following the procedure recommended by the manufacturer to construct the clone library for each xylanase family. Positive transformants (white colonies) from each library were randomly picked for further confirmation by PCR with primers M13F (GTAAAACGACGGCCAGT) and M13R (GGATAACAATTTCACACAGGA). They were then sequenced by Life Technologies using the Sanger method with an ABI-3730 automatic sequencer (Life Technologies, Carlsbad, CA).

### Phylogenetic analysis

By using the Figaro software [28] (http://sourceforge.net/apps/mediawiki/amos/index.php?title=Figaro), vector sequences introduced by automated Sanger sequencing machines were removed. The sequences were analyzed by NCBI BLASTx (version 2.2.29) searches against the GenBank nr database, with an E-score (expect value) cutoff of 10^−10^. Nucleotide sequences identified as xylanase gene fragments were translated into amino acids by EMBOSS Transeq (http://www.ebi.ac.uk/Tools/st/emboss_transeq/). Then, they were aligned with known sequences in the GenBank database at the protein level using ClustalW. Redundant amino acid sequences were removed using Cd-hit [29] with a 95% sequence identity cutoff.

The protein sequence similarities were assessed using the BLASTp program (http://www.ncbi.nlm.nih.gov/BLAST/; until January 15, 2014). Phylogenetic trees were constructed with MEGA 4.1 [30] using the neighbor-joining method [31]. Confidence for tree topologies was estimated by bootstrap values based on 1,000 replicates. A total of 39 and 17 representative sequences were selected and used as references for GH10 and GH11 phylogenetic tree constructions, respectively.

### Abundance analysis

The abundance of each GH family was estimated using the distance-based operational taxonomic unit and richness determination (DOTUR) software [32]. By using PHYLIP software (http://evolution.genetics.washington.edu/phylip.html), distance matrices of the fragment sequences were calculated at the protein level with the default parameters of protdist. Based on UPGMA (average linkage clustering) implemented in DOTUR with default parameters of precision (0.01) and 1000 bootstrap replicates, sequences were then assigned to OTUs.

### Cloning of full-length xylanase gene

**Figure 1:**
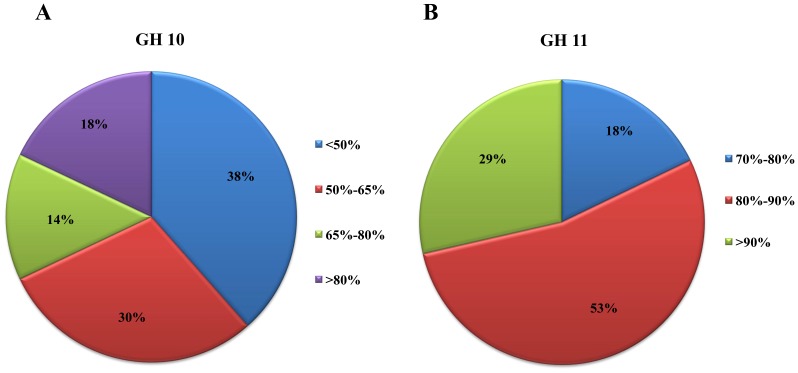
Amino acid sequence homologies of GH10 and GH11 xylanase gene fragments from sediment metagenomic DNA to known xylanases. Each sequence was analyzed by NCBI BLASTp (version 2.2.29) against the GenBank nr database. An E-score (expect value) cutoff of 10^−10^ (default) was applied and the top BLASTp hit to the known xylanases was collected.

Fragment AS10-66, which showed phylogenetic novelty (Figure 1, Table S1) and was most abundant in the GH10 library (see Table S1), was subjected to full-length gene cloning. The flanking regions of the xylanase gene fragments were cloned by using a modified TAIL-PCR with eight specific primers (Table S3) following the protocol [33]. PCR products of the expected size appeared between the third and fourth rounds of amplification were purified, cloned into the PMD 19-T vectors, sequenced, and then assembled with the known fragment sequence. The full-length xylanase gene was designated as *xynAS10-66*. The signal peptide sequence of XynAS10-66 was predicted with SignalP [34] (http://www.cbs.dtu.dk/services/SignalP/). The DNA and protein sequence identities/similarities were assessed with the BLASTn and BLASTp programs (http://www.ncbi.nlm.nih.gov/BLAST/), respectively.

### Xylanase expression and activity assay

Using two primer sets (Table S3), the coding sequence of the xylanase gene *xynAS10-66* without the signal peptide was amplified and cloned into vector pET-22b(+), and then transformed into *E. coli* BL21 (DE3) competent cells for recombinant expression. The positive transformant harboring pET-*xynAS10-66* was grown in LB medium containing 100 µg mL^−1^ ampicillin at 37°C to an A_600_ of 0.6. Protein expression was induced by addition of isopropyl-β-d-1-thiogalactopyranoside (IPTG) at a final concentration of 1 mM, and the culture was incubated at 30°C for additional 12 h.

Xylanase activity was determined by measuring the release of reducing sugar from substrate by the 3, 5-dinitrosalicylic acid method [35]. To accomplish this, reactions containing 0.1 ml of appropriately diluted enzyme and 0.9 ml of 1% (w/v) beechwood xylan as substrate. After incubation at 55°C for 10 min, the reaction was stopped with 1.5 ml DNS reagent and boiled for 5 min, and the absorbance at 540 nm (A_540_) was measured. Finally, using a standard curve generated with d-xylose, the absorbance was converted into moles of reducing sugars produced. One unit (U) of xylanase activity was defined as the amount of enzyme that released 1 µmol of reducing sugar equivalent to xylose per minute.

### Purification and partial characterization of recombinant XynAS10-66

To purify the His-tagged recombinant proteins (rXynAS10-66), culture supernatant was collected after centrifugation (12,000×*g*, 4°C for 15 min). Then the culture supernatant was concentrated with an ultrafiltration membrane (PES5000; Sartorius Stedim Biotech, Germany) and then loaded onto a Ni^2+^-NTA agarose gel column (Qiagen, Germany) with an imidazole gradient of 20–200 mM in Tris-HCl buffer (20 mM Tris-HCl, 500 mM NaCl, 10% glycerol, pH 7.6). Sodium dodecyl sulfate-polyacrylamide gel electrophoresis (SDS-PAGE) was used to determine the purity and apparent molecular mass of rXynAS10-66.

The optimal pH for xylanase activity of the purified rXynAS10-66 was determined at 37°C with pH ranging from 4.0 to 11.0. The buffers used were McIlvaine buffer (0.2 M Na_2_HPO_4_/0.1 M citric acid) for pH 4.0–7.0, 0.1 M Tris-HCl for pH 7.0–9.0, and 0.1 M glycine-NaOH for pH 9.0–11.0. The optimal temperature for purified rXynAS10-66 activity was determined over the range of 40–90°C in Tris-HCl buffer (pH 9.0). The *K*_m_ and *V*_max_ values for rXynAS10-66 were determined in Tris-HCl buffer (pH 7.0) containing 1–10 mg mL^−1^ beechwood xylan at 55°C, respectively. *K*_m_ and *V*_max_ were determined from a Lineweaver-Burk plot using the non-linear regression computer program GraFit (Erithacus, Horley, Surrey, UK).

### Nucleotide sequence accession numbers

The GH10 and GH11 xylanase gene fragments were deposited into the GenBank database under accession numbers KJ463250–KJ463327 and KJ463328–KJ463355, respectively. Accession number KJ463356 was assigned to the full-length xylanase gene *xynAS10-66*.

## Results and Discussion

Soda lakes are one of the most stable naturally occurring alkaline and saline environments that harbor numerous novel microorganisms [16], [17], [21]. Although the microbial diversity of this unique ecosystem has been thoroughly investigated, only a few studies have considered the functional diversity [22], [23]. Functional gene diversity based on the metagenomic sequences can provide insight into functional diversity and metabolic potential at the community level. Xylanases play key roles in the initial steps of plant cell wall breakdown and have great potential for industrial and agricultural applications. Thus xylanase genes were targeted for diversity analysis in this study. Because many microorganisms cannot be cultured in the laboratory owing to the extremely harsh conditions of soda lakes, culture-independent molecular approaches were used to explore the xylanase gene diversity of Lake Dabusu.

### Abundance of GH10 xylanase in Lake Dabusu sediment

Using the CODEHOP primers X10-F and X10-R specific for GH10 xylanases [27], PCR product of about 260 bp was amplified from the metagenomic DNA of the sediment. The product was purified and used to construct a clone library. The positive transformants were picked and then confirmed by PCR with primers M13F and M13R. Overall, 550 clones were sequenced, 467 sequences of which were identified as GH10 xylanase gene fragments based on BLASTx analysis and presence of the Asn residue in the protein sequence, which is conserved among GH10 xylanases [36].

After removing redundant sequences using the CD-hit program [29], 78 sequences showed divergence (<95% homology, Table S1). Based on BLASTp analysis, about 68% (53/78) of the sequences had low similarities (<65%) with known xylanases in GenBank (Figure 1), implying that they may be novel xylanases. Abundance analysis using the distance based operational taxonomic unit and richness determination (DOTUR) software [32] showed that AS10-66 was the predominant operational taxonomic unit (OTU), representing 117 sequences. Forty OTUs contained only one sequence (Table S1).

Culture dependent and culture-independent methods revealed the presence of numerous novel microorganisms in soda lake environments [16], [21]. In the present study, GH10 xylanase gene fragment sequences obtained directly from the sediment metagenomic DNA had low homology with known xylanases. Specifically, none of the xylanase fragments amplified in this study had greater than 90% homology with known xylanases in the GenBank database (Table S1). The lowest similarities were observed for AS10-35 and AS10-88, which both had 35% homology with xylanase from *Nocardiopsis* sp. CNS639 (WP_019610727), while AS10-8 showed the greatest homology of 87% with xylanase from *Cellulophaga algicola* DSM 14237 (YP_004163134). Moreover, almost 70% of the obtained sequences had low similarity (<65%) with known xylanases. This is much higher than that from the other soil environments we previously investigated [24]. Overall, these findings suggested that there may be abundant novel xylan-degrading microorganisms in this environment.

### High genetic diversity of GH10 xylanase in the alkaline soda lake sediment

**Figure 2:**
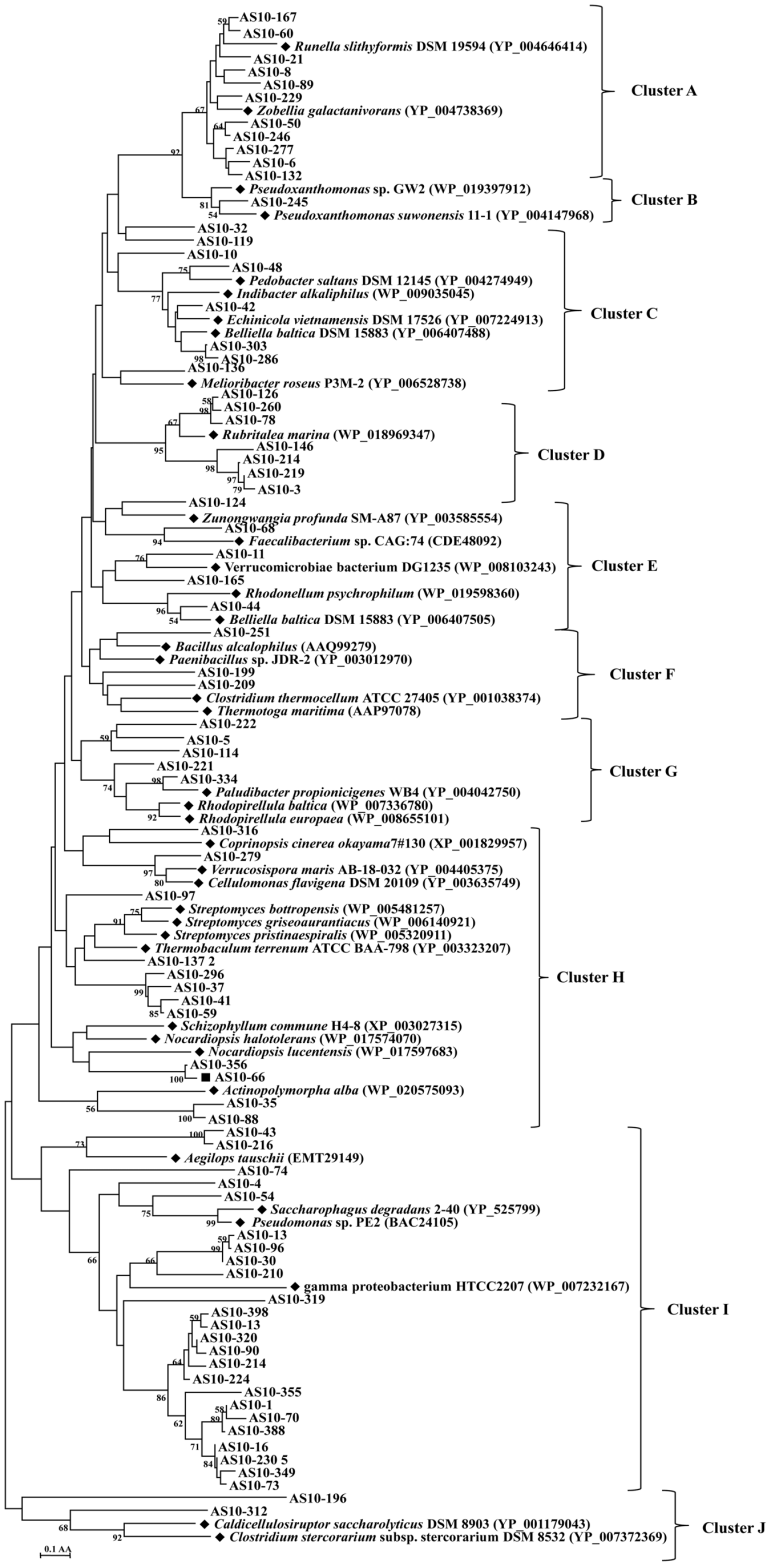
Phylogenetic analysis based on the partial amino acid sequences of GH10 xylanase genes detected in the Lake Dabusu sediment metagenomic DNA. The tree was constructed using the neighbor joining method (MEGA 4.1). The lengths of the branches indicate the relative divergence among amino acid sequences. The reference sequences are marked with a closed diamond (♦) with source strains and GenBank accession numbers in parentheses. The gene fragments (AS10-66) used for full length cloning were marked with a solid square (▪). The numbers at the nodes indicate bootstrap values based on 1,000 bootstrap replications and bootstrap values (>50) are displayed. The scale bar represents 0.1 amino acid substitutions per position.

Using 78 divergent sequences from the GH10 clone library and 39 reference sequences from the GenBank Database, an unrooted protein-level phylogenetic tree of GH10 xylanases was constructed. All sequences were confined to ten clusters, denoted as Cluster A to Cluster J, indicating substantial diversity among GH10 xylanases in the sediment (Figure 2). The presence of many clades without close relatives suggests their novelty, which might be because of the large portion of unidentified microorganisms in this environment.

Cluster A, C, E and G contained a total of 28 sequences from the soda lake sediment and 14 reference sequences of genera belonging to Bacteroidetes including *Runella slithyformis* DSM 19594, *Pedobacter saltans*, *Indibacter alkaliphilus* and *Rhodopirellula baltica*. Cluster B and cluster I contained the sequences of 23 clones and reference sequences from the phylum Proteobacteria, including gamma proteobacterium HTCC2207, *Pseudomonas* sp. PE2, *Saccharophagus degradans* 2–40 and *Pseudoxanthomonas suwonensis* 11-1. Twelve sequences and 11 reference sequences from different genera in the phylum Actinobacteria, including *Actinopolymorpha*, *Streptomyces* and *Nocardiopsis,* formed cluster H. Three sequences in cluster F and two in cluster J were closely related to xylanase produced by members of the phylum Firmicutes, including *Bacillus alcalophilus*, *Paenibacillus* sp. JDR-2, *Clostridium thermocellum* ATCC 27405, *Caldicellulosiruptor saccharolyticus* DSM 8903 and *Clostridium stercorarium* subsp. stercorarium DSM 8532. Seven sequences in cluster D shared the highest identity with xylanase from *Rubritalea marina*.

As shown in Fig. 2, phylogenetic analysis revealed that Bacteroidetes, Proteobacteria, Actinobacteria, Firmicutes and Verrucomicrobia are the main xylanolytic bacteria to produce xylanases in the sediment of Lake Dabusu. These findings are consistent with the conclusions of previous studies [16], [17], [20], [37]. However, the composition of the xylanolytic community differs from that of the microbial community. Microbial community analysis suggested that Firmicutes are the most abundant microorganisms in soda lake environments, followed by Proteobacteria and Actinobacteria. In the present study, xylanases were found to primarily belong to Bacteroidetes, which harbored more than 35% (28/78) of all GH10 fragment sequences. One reason for this may be that the xylantic microbial community differs from that of the total microbial community. Soda lakes harbor abundant and diverse microorganisms which are critical in decomposition of organic matter and cycling of carbon, nitrogen, phosphorus, and sulphur [38]. Second, the physicochemical properties of Lake Dabusu differ from those of other soda lakes [16], which play important role in the composition of the microbial community [20], [39].

Xylanase fragment sequences related to those from Proteobacteria were the second most abundant, accounting for about 30% (23/78) of all GH10 xylanase sequences. All reference sequences of gamma proteobacterium HTCC2207, *Pseudomonas* sp. PE2, *Saccharophagus degradans* 2–40 and *Pseudoxanthomonas suwonensis* 11-1 belonged to the Gammaproteobacteria. This is concurrent with the finding that Gammaproteobacteria is one of the most abundant and diverse groups in soda lakes [37]. Moreover, we found that although these sequences were grouped in a cluster, they were not closely related to each other (Figure 2), suggesting that these sequences might be novel.

### Amplification and sequence analysis of GH11 xylanase gene fragments

PCR product with a size of about 210 bp was obtained from the metagenomic DNA of sediment using CODEHOP primers X11-F and X11-R specific for GH11 xylanases [24]. Overall, 250 clones were randomly selected from the clone library constructed using the PCR products and sequenced. As a result, 204 sequences showed 70–98% amino acid identity with known GH11 xylanases (Table S2). Additionally, Glu (catalytic residue), which is highly conserved in GH11 xylanases, was found in all protein sequences (Table S2). Thus, these sequences were considered to be partial GH11 xylanases. After removing the redundant sequences using the CD-hit program, 28 sequences showed divergence (sharing <95% identity) (Table S2). Abundance analysis using DOTUR software showed that AS11-42 was the predominant OTU, occurring in 39 sequences. Nine OTUs contained only one sequence (Table S2).

### Phylogenetic diversity of GH11 xylanase in lake sediment

**Figure 3:**
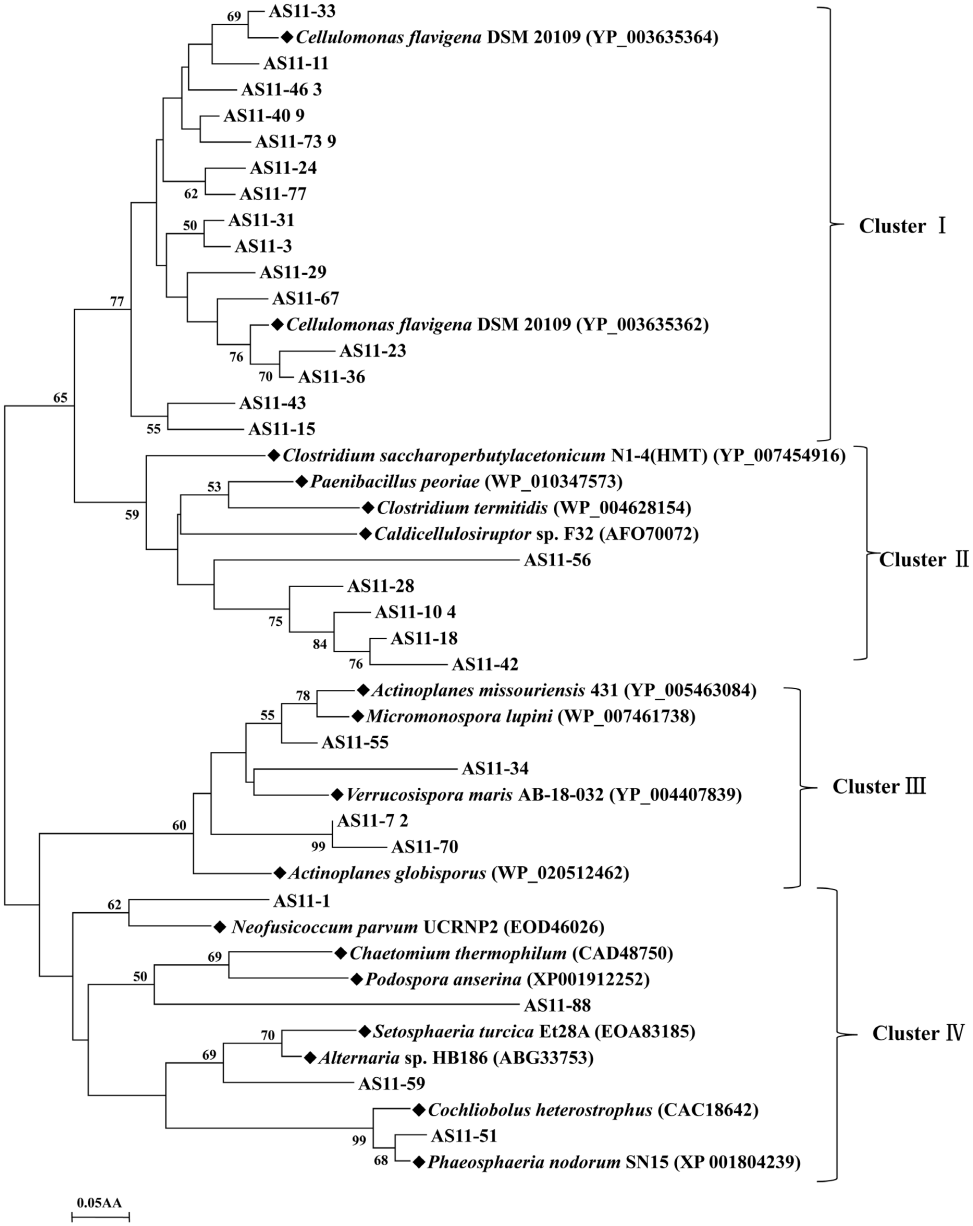
Phylogenetic analysis based on the partial amino acid sequences of GH11 xylanase genes detected in the Lake Dabusu sediment metagenomic DNA. The tree was constructed using the neighbor joining method (MEGA 4.1). The lengths of the branches indicate the relative divergence among amino acid sequences. The reference sequences are marked with a closed diamond (♦) with source strains and GenBank accession numbers in parentheses. The numbers at the nodes indicate bootstrap values based on 1,000 bootstrap replications and bootstrap values (>50) are displayed. The scale bar represents 0.05 amino acid substitutions per position.

The 28 distinct partial sequences of GH11 xylanases were used to construct an unrooted phylogenetic tree with 17 reference sequences (Figure 3). Four clusters (I, II, III, and IV) were formed based on high bootstrap values. Some clades formed without closely related references, suggesting that these sequences differed from known xylanases. Nineteen sequences shared the highest identity with xylanases from *Cellulomonas flavigena* DSM 20109, *Actinoplanes missouriensis* 431, *A. globisporus Micromonospora lupine* and *Verrucosispora maris* AB-18-032, and fell into clusters I and III. Five sequences and those of *Clostridium saccharoperbutylacetonicum* N1-4(HMT), *C. Paenibacillus peoriae*, and *Caldicellulosiruptor* sp. F32 were grouped into cluster II. Four sequences closely related to xylanases from *Neofusicoccum parvum* UCRNP2, *Podospora anserine*, *Setosphaeria turcica* Et28A, *Alternaria* sp. HB186 and *Phaeosphaeria nodorum* SN15 fell into cluster IV.

Unlike GH10 family xylanase genes, GH11 xylanase genes from Lake Dabusu were less diverse and showed a different microbial distribution. Sequences of the GH11 xylanase fragments were found to be related to Actinomycetes, Firmicutes and Fungi. Actinomycetes are important degraders of organic matter in most habitats. An alkaline xylanase was purified and characterized from alkaliphilic *Micrococcus* sp. AR-135, which was isolated from an alkaline soda lake in Ethiopia [14]. Upon phylogenetic analysis, more than 67% (19/28) of the sequences were grouped into a cluster containing reference sequences from different genera in Actinobacteria. Along with the 12 sequences of GH10 xylanase, a total 31 fragment sequences were found to be associated with Actinobacteria, indicating the high diversity of xylanolytic actinobacteria in Lake Dabusu.

Using culture and culture-independent methods, the bacterial community in the soda lake was found to be dominated by clones affiliated with Firmicutes. Moreover, several xylanases were characterized from the isolated Firmicutes with high activity at alkaline pH [13], [15]. In this study, xylanase fragments related to xylanases of different genera Firmicutes were also found. Specifically, five GH10 xylanase fragment sequences were found to be closely related to *Bacillus alcalophilus*, *Clostridium thermocellum* ATCC 27405 and *Caldicellulosiruptor saccharolyticus* DSM 8903, while five GH11 fragments were closely related to *Caldicellulosiruptor* sp. F32 (AFO70072), *Clostridium termitidis* (WP_004628154) and *Clostridium saccharoperbutylacetonicum* N1-4(HMT) (YP_007454916), suggested the diversity of xylanolytic Firmicutes in this environment.

When compared with Bacteria and Archaea, little is known about the diversity, abundance and activity of micro-eukaryotes in soda lakes. Recently, a high diversity of micro-eukaryotes in soda lakes located in the Ethiopian Rift Valley was revealed by high-throughput sequencing [21]. However, no functional genetic diversity has been reported regarding fungi in soda lake environments. In the present study, four sequences were found to be related to xylanases from Ascomycota, the dominant fungi in soda lakes, suggesting that xylantic fungi are present in these systems.

### Direct cloning and novelty of xylanase gene XynAS10-66

Until recently, the majority of known xylanases were obtained from pure cultures. Only a few were cloned using culture-independent approaches, such as construction and screening of metagenomic libraries and PCR-based methods. In our lab, we developed a fast and efficient modified TAIL-PCR method to obtain full-length genes directly from metagenomic DNA based on fragment sequences [33]. In this study, the fragment sequence AS10-66 was selected to obtain the full-length gene because it was the most abundant of all the sequences, representing about one quarter of all sequences (117/467). Moreover, AS10-66 has been reported to have low homology with known xylanases (Table S1).

Based on the fragment sequences of AS10-66 and using modified TAIL-PCR [33], a full-length xylanase gene (*xynAS10-66*) was direct cloned from metagenomic DNA of the soda lake sediment. The complete sequence of *xynAS10-66* contained an open reading frame of 1092 bp encoding a 363-residue polypeptide with a typical signal peptide (residues 1–24). The calculated mass of XynAS10-66 was 38.7 kDa, and the theoretical isoelectric point was 4.74. Sequence similarity searches showed that deduced XynAS10-66 shared the highest homology (44%) with the thermostable GH10 xylanase from *Thermomonospora alba* ULJB1, followed by *Thermobispora bispora* DSM 43833 (44%), an isolate recovered from decaying manure and *Nocardiopsis halotolerans* (43%), an isolate recovered from salt marsh soil in Kuwait [40].

### Partial biochemical characterization of purified recombinant XynAS10-66

**Figure 4:**
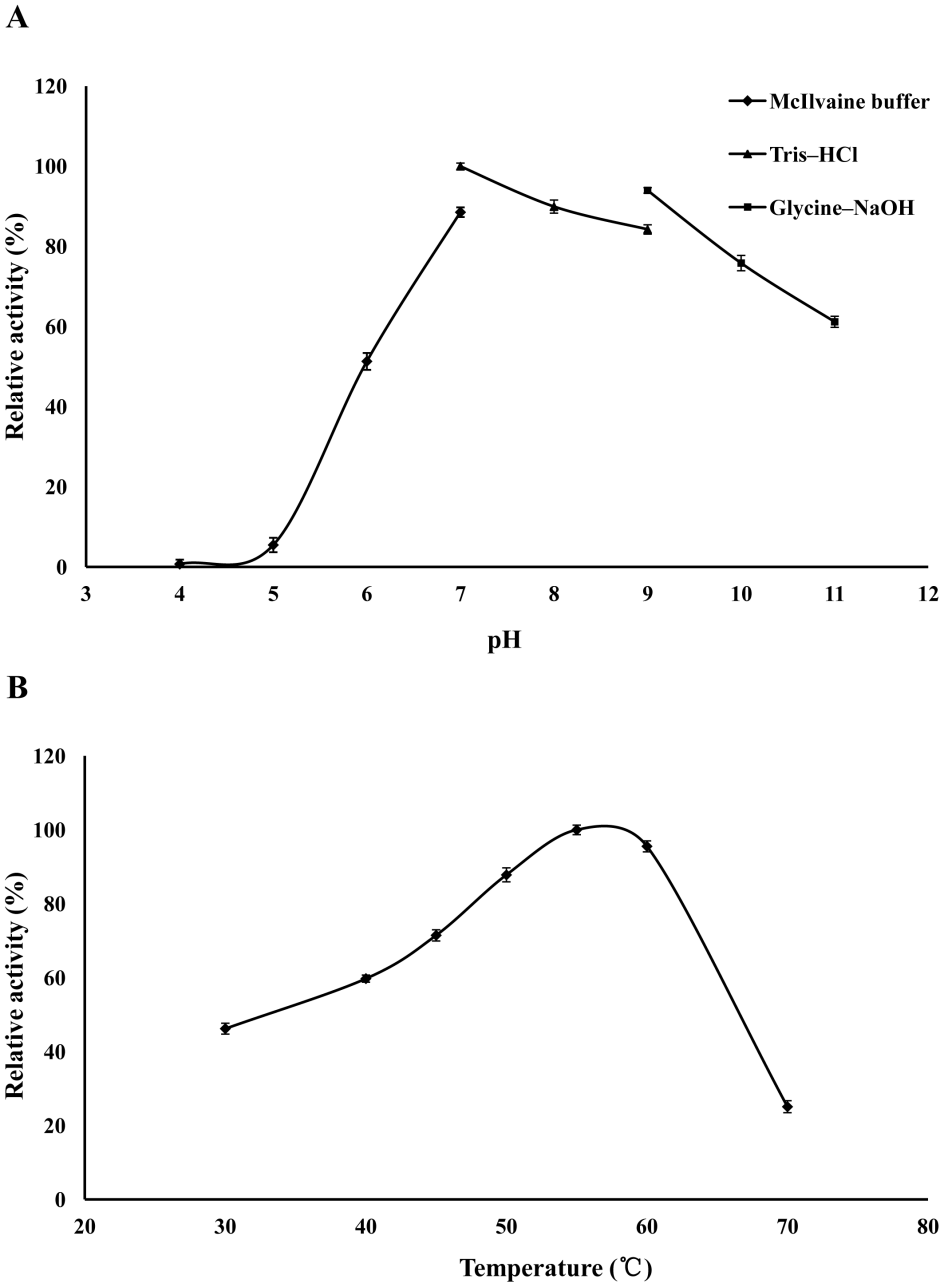
pH and temperature activity profiles of purified recombinant XynAS10-66. **A** Effect of pH on XynAS10-66 activity. Activities at various pHs were assayed at 30°C in different buffer. **B** Effect of temperature on XynAS10-66 activity in Tris-HCl buffer (pH 9.0).

The gene encoding the mature proteins was expressed in *E. coli* BL21 (*DE3*). Following induction with IPTG at 30°C for 12 h, substantial xylanase activity was detected in the culture supernatant of recombinant cells. Using beechwood xylan as the substrate, rXynAS10-66 showed the highest activity at pH 7.0, while it retained more than 95% activity at pH 9.0, and >60% of the maximum activity at pH 7.0–11.0 (Figure 4A). Using beechwood xylan as the substrate, the *K_m_* and *V_max_* values were 1.5±0.04 mg · mL^−1^, 1102±9.54 µmol · mg^−1^ · min^−1^, respectively. Although the optimum temperature of the recombinant XynAS10-66 is neutral, it has substantial activity at alkaline pH, with more than 80% and 60% activity being retained at pH 10 and pH 11, respectively. This characterization differs from those of alkaline xylanases obtained from *Bacillus* and *Micrococcus* sp. isolated from other soda lakes, which showed optimum activity at pH 8 to 9 [14], [15]. The optimal temperature for the enzyme activity of XynAS10-66 was 55°C (Figure 4B), while more than 60% activity was retained at 45°C to 65°C. The recombinant XynAS10-66 had the same optimum temperature as the alkaline xylanase from alkaliphilic *Micrococcus* sp. AR-135 [14]. However, it had a lower activity than that of xylanases from *Bacillus halodurans* S7 [13] and two thermostable alkaline xylanases from an alkaliphilic *Bacillus* sp. [15]. BLAST analysis revealed that XynAS10-66 shared high similarity with a thermostable endo-beta-1,4-xylanase of *Thermomonospora alba* ULJB1 [41], which showed good activity at up to 95°C. Sequence alignment revealed that XynAS10-66 lacks a carbohydrate binding module (CBM) at the C terminal, which may have resulted in the lower temperature optimum of XynAS10-66. Future studies will be conducted to investigate this phenomenon. Moreover, other fragment sequences will be used to generate more full-length genes directly from the metagenomic DNA, and any genes generated will be characterized in subsequent studies.

In conclusion, culture-independent molecular methods led to recovery of abundant GH10 and GH11 xylanase genes from the sediment of the Lake Dabusu. Similarities among these sequences with known xylanases were low, and they were distantly related based on phylogenetic analysis. These results suggest that xylanase gene diversity within Lake Dabusu is high and most of them might be novel. Our study provides new insight into the genetic diversity and distribution of xylanases in soda lake environments and a rapid culture-independent molecular method for the retrieval of the source of xylanase genes with potential industrial applications.

## Supporting Information

Table S1 GH10 xylanase gene fragments detected in the sediment of Lake Dabusu and their closest relative based on amino acid sequence identity and similarity.
(PDF)

Table S2 GH11 xylanase gene fragments detected in the sediment of Lake Dabusu and their closest relative based on amino acid sequence identity and similarity.
(PDF)

Table S3 Primers used for gene cloning and expression.
(PDF)
