# Supplementary material for: High Phylogenetic Diversity of Glycosyl Hydrolase Family 10 and 11 Xylanases in the Sediment of Lake Dabusu in China
Source: PLoS One. 2014 Nov 13;9(11):e112798. doi: 10.1371/journal.pone.0112798 (PMC4231106; doi:10.1371/journal.pone.0112798)
Supplement: Table S1 — GH10 xylanase gene fragments detected in the sediment of Lake Dabusu and their closest relative based on amino acid sequence identity and similarity. (PDF) [file pone.0112798.s001.pdf]

Supplementary Table S1. The GH 10 xylanase gene fragments detected in the Dabusu alkaline lake sediment and their closest relatives based on amino acid sequence identity and similarity.

OTU [a]	Protein size (amino acids)	Identity (%)	Amount of sequences	Closest relative (accession No.)
AS10-1	83	45	70	*Pseudomonas* sp. PE2 (BAC24105)
AS10-70	83	47	14	*Pseudomonas* sp. PE2 (BAC24105)
AS10-388	83	50	56	*Pseudomonas* sp. PE2 (BAC24105)
AS10-4	83	54	2	*Pseudomonas* sp. PE2 (BAC24105)
AS10-16	83	50	14	*Pseudomonas* sp. PE2 (BAC24105)
AS10-230	83	50	5	*Pseudomonas* sp. PE2 (BAC24105)
AS10-73	83	48	5	*Pseudomonas* sp. PE2 (BAC24105)
AS10-355	83	48	1	*Pseudomonas* sp. PE2 (BAC24105)
AS10-398	83	47	1	*Pseudomonas* sp. PE2 (BAC24105)
AS10-320	83	48	4	*Pseudomonas* sp. PE2 (BAC24105)
AS10-13	83	47	5	*Pseudomonas* sp. PE2 (BAC24105)
AS10-90	83	48	4	*Pseudomonas* sp. PE2 (BAC24105)

AS10-224	83	48	4	*Pseudomonas* sp. PE2 (BAC24105)
AS10-214	83	47	4	*Pseudomonas* sp. PE2 (BAC24105)
AS10-13	83	45	2	*Pseudomonas* sp. PE2 (BAC24105)
AS10-96	78	48	1	*Pseudomonas* sp. PE2 (BAC24105)
AS10-30	83	45	5	*Pseudomonas* sp. PE2 (BAC24105)
AS10-210	83	42	9	*Pseudomonas* sp. PE2 (BAC24105)
AS10-349	83	49	5	*Actinopolymorpha alba (*WP_020575093)
AS10-219	83	48	1	*Candidatus Solibacter usitatus* Ellin6076 (YP_824087)
AS10-136	84	68	13	*Candidatus Solibacter usitatus* Ellin6076 (YP_823955)
AS10-54	83	63	2	*Teredinibacter turnerae* (WP_019604745)
AS10-43	83	42	1	*Aegilops tauschii* (EMT29149)
AS10-216	83	42	10	*Hordeum vulgare subsp. vulgare* (BAJ92092)
AS10-199	87	56	1	*Clostridium thermocellum* DSM 1313 (YP_005689086)
AS10-209	84	56	1	*Thermotoga naphthophila* RKU-10 (YP_003346209)
AS10-59	85	64	1	*Streptomyces griseoaurantiacus* (WP_006140921)

AS10-41	85	64	1	*Actinoplanes sp. SE50/110* (YP_006263460)
AS10-37	85	68	1	*Streptomyces ipomoeae* (WP_009327081)
AS10-296	85	64	1	*Streptomyces griseoaurantiacus* (WP_006140921)
AS10-137	81	69	2	*Thermobaculum terrenum* ATCC BAA-798 (YP_003323207)
AS10-124	87	59	1	*Zunongwangia profunda* SM-A87 (YP_003585554)
AS10-97	82	57	1	*Paenibacillus fonticola* (WP_019637493)
AS10-221	92	80	1	*Rhodopirellula baltica* (WP_007336780)
AS10-114	93	47	4	*R. europaea* (WP_008655101)
AS10-222	87	48	3	*Haliscomenobacter hydrossis* DSM 1100 (YP_004446188)
AS10-11	86	57	1	Verrucomicrobiae bacterium DG1235 (WP_008103243)
AS10-5	87	42	1	*Chitinophaga pinensis* DSM 2588 (YP_003123895)
AS10-334	94	82	1	*Paludibacter propionicigenes* WB4 (YP_004042750)
AS10-279	96	82	1	*Verrucosispora maris* AB-18-032 (YP_004405375)
AS10-251	86	60	1	*Amphibacillus xylanus* NBRC 15112 (YP_006844174)
AS10-316	85	55	1	*Coprinopsis cinerea okayama*7#130 (XP_001829958)

AS10-356	91	52	1	*Schizophyllum commune H4-8* (XP_003027315)
AS10-66	91	50	117	*Thermobifida alba* (XP_003027315)
AS10-44	84	79	1	*Belliella baltica* DSM 15883 (YP_006407505)
AS10-165	85	55	1	*Bacillus sp.* (CAA84631)
AS10-277	86	86	1	*Cellulophaga algicola* DSM 14237 (YP_004163134)
AS10-21	86	80	2	*Runella slithyformis* DSM 19594 (YP_004646414)
AS10-50	86	83	1	Verrucomicrobiae bacterium DG1235 (WP_008098872)
AS10-245	87	81	1	*Pseudoxanthomonas suwonensis* 11-1 (YP_004147968)
AS10-8	86	87	3	*Cellulophaga algicola* DSM 14237 (YP_004163134)
AS10-89	86	80	1	Verrucomicrobiae bacterium DG1235 (WP_008098872)
AS10-6	86	86	2	*Cellulophaga algicola* DSM 14237 (YP_004163134)
AS10-132	86	84	1	*C. algicola* DSM 14237 (YP_004163134)
AS10-167	86	86	1	*C. algicola* DSM 14237 (YP_004163134)
AS10-60	86	81	7	*Zobellia galactanivorans* (YP_004738369)
AS10-32	84	61	2	*Z. galactanivorans* (YP_004738369)

AS10-229	86	86	1	Verrucomicrobiae bacterium DG1235 (WP_008098872)
AS10-246	86	80	10	Verrucomicrobiae bacterium DG1235 (WP_008098872)
AS10-303	84	83	5	*Marinilabilia salmonicolor* (WP_010663023)
AS10-286	84	82	8	*M. salmonicolor* (WP_010663023)
AS10-42	84	82	2	*Anaerophaga thermohalophila* (WP_016778704)
AS10-48	84	79	1	*Pedobacter saltans* DSM 12145 (YP_004274949)
AS10-119	84	57	2	*Belliella baltica DSM 15883* (YP_006407488)
AS10-126	85	57	2	Verrucomicrobiae bacterium DG1235 (WP_008103539)
AS10-78	85	75	2	*Rubritalea marina* (WP_018969347)
AS10-260	85	72	1	*R. marina* (WP_018969347)
AS10-319	84	63	1	*R. marina* (WP_018969347)
AS10-414	84	64	1	*R. marina* (WP_018969347)
AS10-3	84	63	1	*R.marina* (WP_018969347)
AS10-14	84	58	1	*R. marina* (WP_018969347)
AS10-68	85	58	1	*Faecalibacterium* sp. CAG:74 (CDE48092)

AS10-10	84	60	1	*Pontibacter roseus* (WP_018479478)
AS10-35	91	35	1	*Nocardiopsis* sp. CNS639 (WP_019610727)
AS10-88	91	35	2	*Nocardiopsis* sp. CNS639 (WP_019610727)
AS10-74	82	44	2	*Candidatus Solibacter usitatus* Ellin6076 (YP_824087)
AS10-312	88	41	1	*candidate division* EM 19 (WP_018195926)
AS10-196	83	36	17	*c. division* EM 19 (WP_018195926)
Total 78			467	

[a] Sequence name was selected to represent each OTU.
