# Supplementary material for: High Phylogenetic Diversity of Glycosyl Hydrolase Family 10 and 11 Xylanases in the Sediment of Lake Dabusu in China
Source: PLoS One. 2014 Nov 13;9(11):e112798. doi: 10.1371/journal.pone.0112798 (PMC4231106; doi:10.1371/journal.pone.0112798)
Supplement: Table S2 — GH11 xylanase gene fragments detected in the sediment of Lake Dabusu and their closest relative based on amino acid sequence identity and similarity. (PDF) [file pone.0112798.s002.pdf]

Supplementary Table S1. The GH 11 xylanase gene fragments detected in the Dabusu alkaline lake sediment and their closest relatives based on amino acid sequence identity and similarity.

OTU [a]	Protein size (amino acids)	Identity (%)	Amount of sequences	Closest relative (accession No.)
AS11-24	70	88	22	*Xylanimonas cellulosilytica* DSM 15894 (YP_003325339)
AS11-77	70	90	7	*X. cellulosilytica* DSM 15894 (YP_003325339)
AS11-22	70	87	21	*Cellulomonas flavigena* DSM 20109 (YP_003635363)
AS11-46	69	88	3	*C. flavigena* DSM 20109 (YP_003635363)
AS11-11	69	90	1	*C. flavigena* DSM 20109 (YP_003635363)
AS11-40	69	88	19	*C. flavigena* DSM 20109 (YP_003635363)
AS11-73	69	86	14	*C. flavigena* DSM 20109 (YP_003635363)
AS11-31	70	92	1	*C. flavigena* DSM 20109 (YP_003635363)
AS11-3	70	92	9	*C. flavigena* DSM 20109 (YP_003635362)
AS11-67	70	92	1	*C. flavigena* DSM 20109 (YP_003635362)
AS11-33	70	96	2	*C. flavigena* DSM 20109 (YP_003635364)
AS11-23	70	94	2	*C. flavigena* DSM 20109 (YP_003635363)

AS11-36	70	98	9	*C. flavigena* DSM 20109 (YP_003635363)
AS11-43	69	80	1	*Streptomyces costaricanus* (ADU25496)
AS11-15	69	82	1	*S. costaricanus* (ADU25496)
AS11-56	70	70	1	*Clostridium saccharoperbutylacetonicum (YP_007454916)*
AS11-28	70	85	2	*Sorangium cellulosum* So0157-2 (YP_008155261)
AS11-10	70	85	4	*S. cellulosum* So0157-2 (YP_008155261)
AS11-18	70	81	1	*S. cellulosum* So0157-2 (YP_008155261)
AS11-42	69	75	29	*Caldicellulosiruptor kronotskyensis* 2002 (YP_004022816)
AS11-88	79	70	9	*Chaetomium thermophilum* (CAD48750)
AS11-1	71	85	1	*Neofusicoccum parvum* UCRNP2 (EOD46026)
AS11-59	71	83	1	*Setosphaeria turcica* Et28A (EOA83185)
AS11-51	71	96	1	*Cochliobolus heterostrophus* (CAC18642)
AS11-34	71	77	1	*Verrucosispora maris* AB-18-032 (YP_004407839)
AS11-55	71	96	1	*Actinoplanes missouriensis* 431 (YP_005463084)
AS11-7	71	85	2	*Actinoplanes globisporus* (WP_020512462)

AS11-70	71	81	1	*A. globisporus* (WP_020512462)
Total 28			204	

[a] Sequence name was selected to represent each OTU.
