# Supplementary material for: High Phylogenetic Diversity of Glycosyl Hydrolase Family 10 and 11 Xylanases in the Sediment of Lake Dabusu in China
Source: PLoS One. 2014 Nov 13;9(11):e112798. doi: 10.1371/journal.pone.0112798 (PMC4231106; doi:10.1371/journal.pone.0112798)
Supplement: Table S3 — Primers used for gene cloning and expression. (PDF) [file pone.0112798.s003.pdf]

Primers	Sequences (5'→3') [a]	Size (bp)
**AS10-66-uSP1**	CAAGTTCTTGTGCCATCTGGTAGACCCGATCTG	33
**AS10-66-uSP2**	CCGATCTGCCTTGGCCATAGTCCACGTGGTGC	32
**AS10-66-uSP3**	GCCGCCGTTGATGTTGAAGTCGTTATAGTAGAG	33
**AS10-66-uSP4**	CAGATTGATTCATTGCGATGGGTAACC	27
**AS10-66-dSP1**	GGAATCCGGTTACCCATCGCAATGAATCAATCTGG	35
**AS10-66-dSP2**	CCATCGCAATGAATCAATCTGGTATACGACCATC	34
**AS10-66-dSP3**	CCATCGGTAACTCCTACATTGCAGAAGCCTTCAC	34
**AS10-66-dSP4**	CACCACGTGGACTATGGCCAAGGCAGATC	29
**AS10-66-m-F**	GAA**CCATGG**ATTGCGGCGAAACCCCGGTTTACG	33
**AS10-66-m-R**	GGA**GCGGCCGC**CTCTACCCATTCCTCAAGGACTGC	35
